# The Binding of Monomeric C-Reactive Protein (mCRP) to Integrins αvβ3 and α4β1 Is Related to Its Pro-Inflammatory Action

**DOI:** 10.1371/journal.pone.0093738

**Published:** 2014-04-02

**Authors:** Masaaki Fujita, Yoko K. Takada, Yoshihiro Izumiya, Yoshikazu Takada

**Affiliations:** Departments of Dermatology, and Biochemistry and Molecular Medicine, University of California Davis School of Medicine, Sacramento, California, United States of America; Lerner Research Institute, United States of America

## Abstract

The prototypic acute phase reactant C-reactive protein (CRP) is not only a marker but also a potential contributor to inflammatory diseases. CRP exists as the circulating native, pentameric CRP (pCRP) and the monomeric isoform (mCRP), formed as a result of a dissociation process of pCRP. mCRP is highly pro-inflammatory, but pCRP is not. The mechanism of pro-inflammatory action of mCRP is unclear. We studied the role of integrins in pro-inflammatory action of mCRP. Docking simulation of interaction between mCRP and integrin αvβ3 predicted that mCRP binds to αvβ3 well. We found that mCRP actually bound to integrins αvβ3 and α4β1 well. Antagonists to αvβ3 or α4β1 effectively suppressed the interaction, suggesting that the interaction is specific. Using an integrin β1 mutant (β1-3-1) that has a small fragment from the ligand binding site of β3, we showed that mCRP bound to the classical RGD-binding site in αvβ3. We studied the role of integrins in CRP signaling in monocytic U937 cells. Integrins αvβ3 and α4β1 specifically mediated binding of mCRP to U937 cells. mCRP induced AKT phosphorylation, but not ERK1/2 phosphorylation, in U937 cells. Notably, mCRP induced robust chemotaxis in U937 cells, and antagonists to integrins αvβ3 and α4β1 and an inhibitor to phosphatidylinositide 3-kinase, but not an MEK inhibitor, effectively suppressed mCRP-induced chemotaxis in U937 cells. These results suggest that the integrin and AKT/phosphatidylinositide 3-kinase pathways play a role in pro-inflammatory action of mCRP in U937 cells. In contrast, pCRP is predicted to have a limited access to αvβ3 due to steric hindrance in the simulation. Consistent with the prediction, pCRP was much less effective in integrin binding, chemotaxis, or AKT phosphorylation. These findings suggest that the ability of CRP isoforms to bind to the integrins is related to their pro-inflammatory action.

## Introduction

The prototypic acute phase reactant C-reactive protein (CRP) belongs to the family of pentraxins and consists of five identical non-covalently linked subunits. Plasma CRP levels increase during inflammatory states, a property that has long been utilized for clinical purposes. Recent evidence suggests that CRP is not only a marker but also a potential contributor to inflammatory diseases [1]–[3]. Recently, several prospective clinical studies have shown independently that modest elevations in baseline CRP levels predict future cardiovascular events [4]–[7]. CRP is present as two conformations: the circulating native, pentameric CRP (pCRP) and the monomeric or modified CRP (mCRP), formed as a result of a dissociation process of pCRP. In vitro both isoforms exhibit a very distinct inflammatory profile [1]. mCRP is a strongly pro-inflammatory protein, but pCRP is not [1]. There is a localized, physiologically relevant pCRP dissociation mechanism by activated platelets and apoptotic cells and mCRP deposits in inflamed tissues [3]. mCRP binds to endothelial cells, neutrophils, and macrophages [1]. However, the receptors for mCRP have not been fully established. CD16 has been identified as a receptor for mCRP in neutrophils [8], this receptor does not seem to be a major mediator of mCRP's action in endothelial cells [9] or in platelets [10].

Integrins are a family of cell adhesion receptors that recognize extracellular matrix ligands, cell surface ligands, and soluble ligands (such as growth factors) [11], [12]. Integrins are transmembrane αβ heterodimers, and at least 18 α and 8 β subunits are known [12]. Integrins are involved in signal transduction upon ligand binding, and their functions are in turn regulated by signals from within the cell [11]. It has been reported that platelets adhere to pCRP through integrin αIIbβ3 and this interaction is involved in pCRP-mediated suppression of platelet aggregation [13]. However, integrin αIIbβ3 is expressed only in platelets and it is unclear if this integrin binds to mCRP.

In the present study, we studied if integrins are involved in the binding of CRP isoforms and their mechanism of action. We performed docking simulation of interaction between integrin αvβ3 and mCRP. The simulation predicts that mCRP binds to αvβ3, but pCRP does not due to steric hindrance. Consistently we found that mCRP, and less effectively pCRP, bound to αvβ3 and another integrin α4β1. Both integrins play a role in binding of mCRP to monocytic U937 cells. mCRP, but not pCRP, robustly induced chemotaxis in an integrin-dependent manner, and induced AKT phosphorylation in U937 cells. These finding suggests that the ability of mCRP to bind to the integrins is related to its pro-inflammatory action.

## Materials and Methods

### Materials

We used commercially available human pCRP (Lee BioSolutions, St Louis, MO, synthesized in E.Coli). pCRP was stored in 10 mM Tris-HCl (pH 7.5) with 2 mM CaCl_2_ to prevent spontaneous formation of mCRP from pCRP. mCRP was prepared by treating pCRP with 8 M urea/10 mM EDTA for 1 h at 37°C as described [14], [15]. We did not detect endotoxin in the pCRP used in this study using endotoxin detection kit (Pierce LAL Chromogenic Endotoxin Quantitation Kit, Thermo Scientific) (data not shown). mAb 7E3 (anti-human integrin β3) and mAb AIIB2 (anti-human integrin β1) hybridomas were obtained from ATCC. mAb SG73 (anti-human α4) hybridoma was a kind gift from K. Miyake (University of Tokyo). Anti-phospho-AKT (Thr-308), anti-phospho-ERK1/2, anti-ERK1/2, anti-AKT were purchased from Cell Signaling Technology, Inc. (Danvers, MA). Cyclic RGDfV [16] was purchased from Enzo Life Sciences (Plymouth Meeting, PA). BIO1211 was obtained from Tocris Bioscience (Ellisville, MO). LY294002 and PD98059 were purchased from Promega (Madison, WI). Chinese hamster ovary (CHO) cells that express WT β1, β3, or the β1–3-1 mutant have been described [17]. CHO cells that express human α4 have been described [18]. Recombinant soluble αvβ3 has been described [19].

### Methods

#### Cell adhesion assays

Cell adhesion assays were performed as described [17]. Briefly, wells of 96-well Immulon-2 microtiter plates were coated with 100 μl of PBS (10 mM phosphate buffer, 0.15 M NaCl, pH 7.4) containing mCRP or pCRP for 2 h at 37°C, and the remaining protein binding sites were blocked by incubating with 0.1% bovine serum albumin for 1 h at room temperature. Cells (10^5^ cells/well) in 100 μl of adhesion buffer (Hepes-Tyrode's buffer or RPMI1640) were added to the wells and incubated at 37°C for 1 h. After gently rinsing the wells three times with the same buffer to remove unbound cells, bound cells were quantified using endogenous phosphatase Activity [17].

#### Binding assays

ELISA-type soluble integrin binding assays were performed as described [20]. Briefly, wells of 96-well microtiter plate were coated with mCRP or pCRP and the remaining protein-binding sites were blocked as described for adhesion assays. Soluble recombinant integrin αvβ3 in 50 μl in Hepes-Tyrode's buffer supplemented with cations was added to the wells and incubated at room temperature for 2 h. Then non-bound soluble integrin was removed by rinsing the wells with the same buffer. Horseradish peroxidase (HRP)-conjugated anti-His tag mouse IgG was added to the wells and incubated for 60 min. Non-bound antibodies were removed by rinsing the wells with the same buffer, and bound integrins were quantified by measuring the absorbance at 450 nm developed from adding the substrate 3,3,5,5 -tetramethylbenzidine of HRP.

#### Signaling Assays

Serum-starved U937 cells were incubated with CRP isoforms (100 μg/ml) for 5 to 30 min at 37°C. We analyzed cell lysates by Western blotting using specific antibodies. Bound IgG was detected using HRP-conjugated second antibody and SuperSignal West Pico chemiluminescent substrate (Thermo Scientific). We analyzed images using a Fuji LAS 4000mini luminescent image analyzer and MultiGauge V3.0 software (Fujifilm, Tokyo, Japan).

#### Chemotaxis

Chemotaxis was measured in modified Boyden chambers (Transwells). 50 μg/ml mCRP or pCRP in 600 μl RPMI 1640 medium was placed in the lower chamber, and U937 cells (5×10^5^ cells per well) were placed in the upper chamber. In some case, U937 cells were preincubated with antibodies (25 μg/ml mouse IgG, 7E3, SG73 and AIIB2) or inhibitors (50 μM PD98059 and LY294002) for 30 min at 37°C. After 4 h incubation at 37°C, cells in the lower chamber was counted.

Treatment differences were tested using ANOVA and Tukey's multiple comparison test to control the global type I error using Prism 5.0a (Graphpad Software).

Docking simulation was performed as previously described [19], [20] using AutoDock3 and ADT [21].

## Results and Discussion

### Docking simulation predicts that mCRP binds to αvβ3

**Figure 1:**
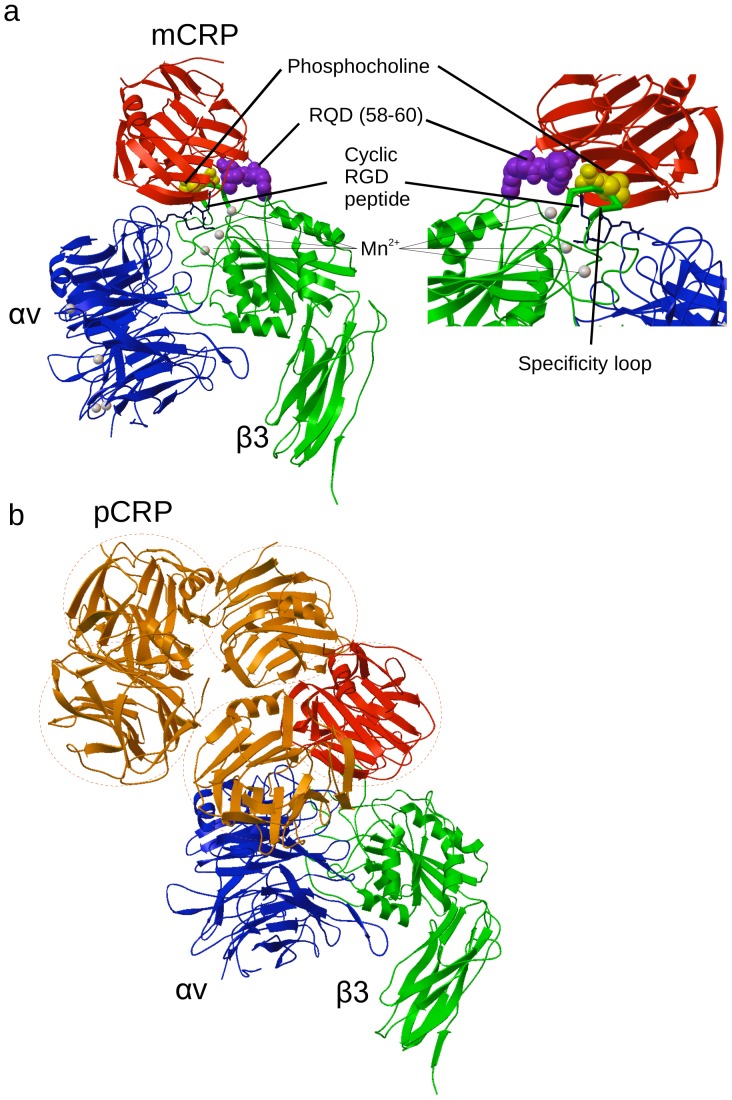
Docking simulation predicts that mCRP binds to integrin αvβ3 but pCRP does not. a) The headpiece of integrin αvβ3 (PDB code 1LG5) was used as a target. The docking model predicts that mCRP (red) binds to the RGD-binding site of the integrin αvβ3 headpiece (green and blue). Amino acid residues involved in αvβ3-mCRP interaction are in Table 1. Cations (Mn) and cyclic RGD peptide and specificity loop of β3 are close to the predicted mCRP-binding site in integrin αvβ3. Cations and cyclic RGD peptide in 1LG5 were removed during docking simulation. The predicted integrin-binding site in mCRP is also close to the phosphocholine-binding site and the RQD motif in mCRP. b) To check if pCRP binds to the integrin, we superposed the pentameric CRP (pCRP, orange and red) to the bound mCRP (red). Interestingly, there are steric clashes between pentameric CRP and αvβ3. This predicts that pentameric CRP can not fully access to mCRP-binding site in integrins due to steric hindrance.

It has been reported that mCRP binds to endothelial cells, neutrophils, and macrophages. While it has been proposed that CD16 is a receptor for mCRP [8], CD16 is expressed in neutrophils, NK cells, and macrophages, but not expressed in endothelial cells. There should be other receptors for mCRP. Integrin αvβ3 are abundantly expressed in endothelial cells, macrophages, and smooth muscle cells in atherosclerotic area [22]. We hypothesized that integrin αvβ3 is potential receptor for CRP. To test this possibility, we performed docking simulation of interaction between αvβ3 (PDB code 1LG5) [23] and mCRP (a single CRP domain (domain a) taken from the pCRP structure, PDB code 1B09) using AutoDock 3. The simulation predicts that mCRP binds to the RGD-binding site of the integrin αvβ3 headpiece (docking energy −18.8 kcal/mol) (The position of cyclic RGD peptide in 1L5G is shown in Figure 1a). Notably amino acid residues in integrin αv (e.g., Tyr178) and β3 (e.g., the cation-binding sites and in the specificity loop) (Table 1), which are commonly involved in ligand binding, are close to the predicted mCRP-binding site (Figure 1a). This suggests that mCRP binds to a site common to other known ligands. The predicted integrin-binding site in mCRP overlaps with or is close to the phosphocholine-binding site of CRP (Figure 1a). The RGD-like RQD motif (residues 58–60) of CRP, a potential binding site for αIIbβ3 [13], is located close to the αvβ3-binding site, while it is unclear if this motif is involved in integrin binding. We obtained similar docked poses for another human mCRP structure (PDB code 1GNH) (−19.3 kcal/mol) (data not shown).

**Table 1:** Amino acid residues involved in mCRP-αvβ3 interaction.

CRP	αV	β3
Lys57, Arg58, Gln59, Asn61, Glu62, Phe66, Ser68, Lys69, Asp70, Ile71, Ser74, Thr76, Asp77, Gly78, Gly79, Ser80, Glu81, Ile82, Leu83, Phe84, Glu85,Arg118, Lys119, Ser120, Leu121, Lys122, Lys123, Gly124, Tyr125, Thr126, Glu138, Asp140, Glu147, Gln150	Met118, Lys119, Asp146, Ile147, Asp148, Asp150, Gly151, Tyr178, Gln214, Ala215, Ile216	Tyr122, Ser123, Met125, Asp126, Asp127, Leu128, Trp129, Tyr166, Asp251, Asp179, Met180, Lys181, Thr182, Glu312, Asn313, Val314, Ser334, Met335, Asp336, Ser337, Ser338

Amino acid residues in integrin αvβ3 and mCRP within 6 Å to each other in the docking model were identified using Swiss-pdb viewer v. 4.1.

To check if pCRP binds to the integrin, we superposed the pentameric CRP (pCRP, 1B09.pdb) to the bound mCRP (Figure 1b). Interestingly, there are steric clashes between one CRP domain of pCRP and αvβ3, and the model predicts that αvβ3 may not fully access to the predicted integrin-binding site in pCRP due to steric hindrance.

### Specific binding of mCRP to αvβ3

**Figure 2:**
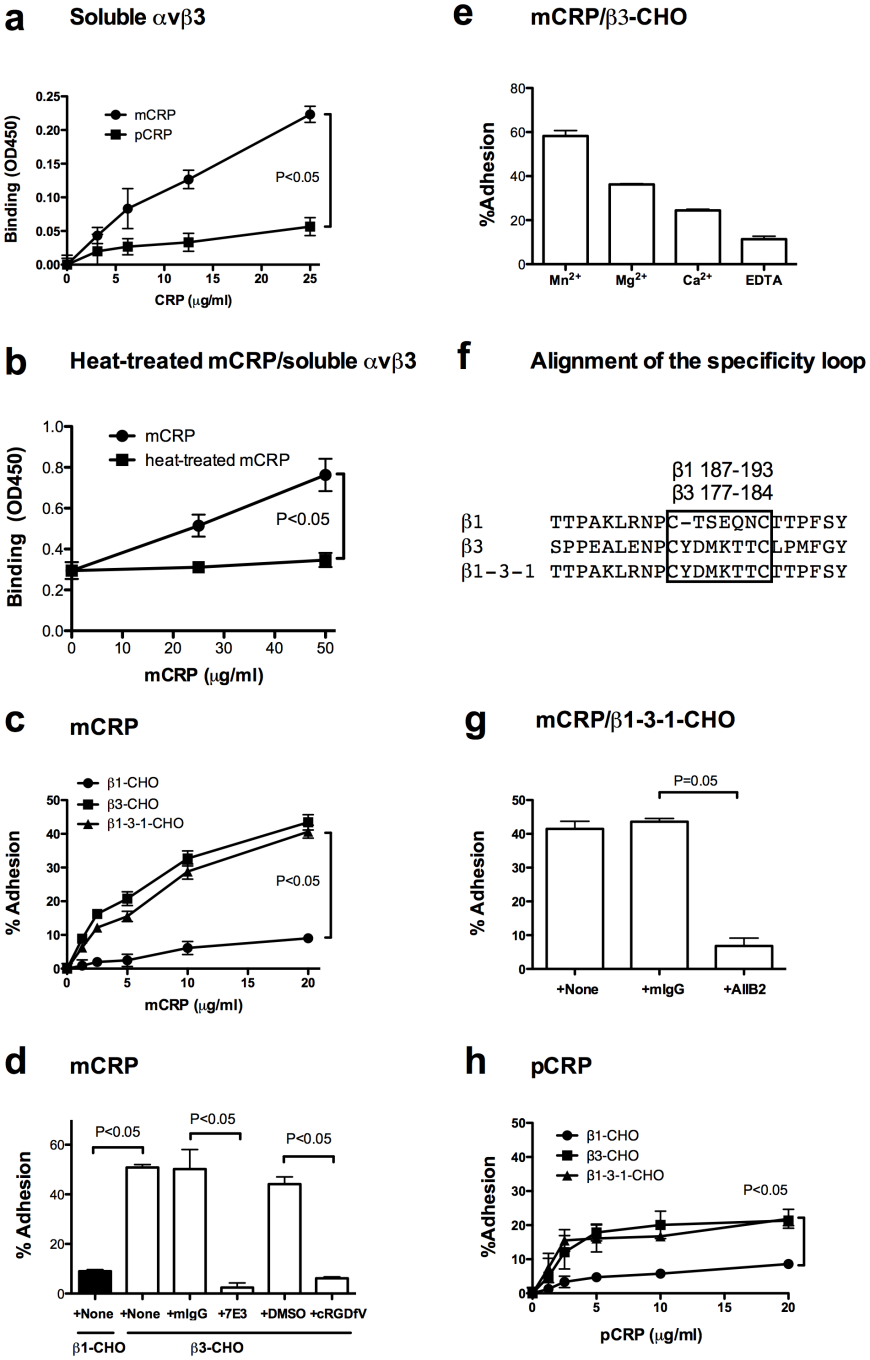
mCRP specifically binds to αvβ3, but pCRP less efficiently binds to the integrin. Wells of 96 well microtiter plate were coated with mCRP or pCRP and remaining protein-binding sites were blocked with BSA. a) ELISA-type integrin binding assay. In a) wells were incubated with recombinant soluble αvβ3 for 2 h at 37°C in Tyrode-HEPES buffer with 1 mM MgCl_2_. b) Effect of heat treatment on mCRP binding to soluble αvβ3. We heated (90°C for 20 min) mCRP before coating wells and used for binding assays. Assays was performed as in a). c) β3- and β1-3-1-CHO cells adhere to mCRP, but β1-CHO cells did not adhere well to mCRP. mCRP was incubated with β1-CHO, β3-CHO, or β1-3-1-CHO cells for 1 h at 37°C in Tyrode-HEPES buffer with 1 mM MgCl_2_. Bound cells were quantified. d) Specificity of αvβ3 binding to mCRP. We tested if inhibitors of αvβ3 block adhesion of β3-CHO cells to mCRP. mCRP was incubated with cells for 1 h at 37°C in Tyrode-HEPES buffer with 1 mM MgCl_2_. mAb 7E3 (to human β3, 10 μg/ml) and cyclic RGDfV (specific antagonist to αvβ3, 10 μM) blocked the adhesion of β3-CHO cells to mCRP, but control purified mouse IgG (mIgG) or vehicle DMSO did not. e) Cation dependency of mCRP binding to αvβ3. Adhesion assays were performed as described in c). mCRP was incubated with β3-CHO cells for 1 h at 37°C in Tyrode-HEPES buffer with 2 mM cations or EDTA. The coating concentration of mCRP is 50 μg/ml. The levels of adhesion in different cation conditions are statistically different. f) Alignment of β1, β3, and β1-3-1 [17]. g) Specificity of β1-3-1 integrin binding to mCRP. We tested if anti-human β1 mAb AIIB2 blocks β1-3-1-CHO cells adhesion to mCRP. (Note: 99% of β1-3-1 is β1 and mAb AIIB2 binds to β1-3-1 and blocks its function). h) Cell adhesion to pCRP. pCRP was incubated with β1-CHO, β3-CHO, or β1-3-1-CHO cells for 1 h at 37°C in Tyrode-HEPES buffer with 1 mM MgCl_2_. Bound cells were quantified.

We tested the prediction using binding assays. We found that immobilized mCRP bound to recombinant soluble integrin αvβ3 in ELISA-type binding assays, but pCRP was much less effective in binding of to αvβ3 (Figure 2a). We also found that heat treatment of mCRP suppressed the binding of soluble αvβ3 to mCRP (Figure 2b). This finding suggests that the binding of mCRP to αvβ3 requires proper folding of mCRP.

We found that mCRP supports adhesion of CHO cells that express recombinant human β3 as hamster αv/human β3 chimera (β3-CHO cells) or those expressing the β1-3-1 mutant (shown below) in a concentration-dependent manner, but did not support adhesion of control CHO cells that express human β1 (β1-CHO) well (Figure 2c). mAb 7E3 (specific to human β3) and cyclic RGDfV peptide (a specific inhibitor of αvβ3) effectively suppressed the adhesion (Figure 2d). These finding suggest that mCRP specifically binds to αvβ3 on the cell surface, and that the classical RGD-binding site of αvβ3 is involved, as predicted. Parent and β1-CHO cells express α5β1, αvβ1, and αvβ5 [17] and we showed that β1-CHO cells do not adhere to mCRP in the present study. It is thus highly likely that these integrins do not bind to mCRP very well.

We also found that the adhesion of β3-CHO cells to mCRP was affected by cations (Mn^2+^>Mg^2+^>Ca^2+^>EDTA) (Figure 2e), suggesting that mCRP is a cation-dependent ligand of αvβ3.

### The specificity loop of β3 is involved in mCRP binding

The integrin β subunit possesses an I-like domain that plays a critical role in ligand binding [12]. We have shown that when a disulfide-linked five-residue sequence of β1 I-like domain (residues 177–183) of αvβ1 is switched with a corresponding sequence in β3 integrin (designated the β1-3-1 mutant) (Figure 2f), ligand-binding specificity of the mutated integrin αvβ1-3-1 is altered to that of αvβ3 [17]. Hence the loop was designated “the specificity loop”. The β1-3-1 mutant (as αvβ1-3-1) bound to vitronectin and fibrinogen, but wt β1 (as αvβ1) did not [17]. The crystal structure of αvβ3 shows that the specificity loop is located in the classical RGD-binding site and undergoes marked conformational changes (1 Å shift) upon RGD binding to αvβ3 [23]. We found that β1-3-1-CHO adhered to mCRP in a concentration-dependent manner like β3-CHO cells (Figure 2c). Anti-β1 mAb effectively suppressed the adhesion of β1-3-1-CHO cells to mCRP. This is consistent with the fact that β1-3-1 is >99% β1 (Figure 2g). These results suggest that the specificity loop of β3 is involved in recognition of mCRP. In contrast, β3- and β1-3-1-CHO cells did not bind to pCRP very well, and we did not test the specificity of interaction using integrin antagonists (Figure 2h). This is consistent with the prediction that αvβ3 has limited access to the integrin-binding site of pCRP.

### Integrin α4β1 binds to mCRP

**Figure 3:**
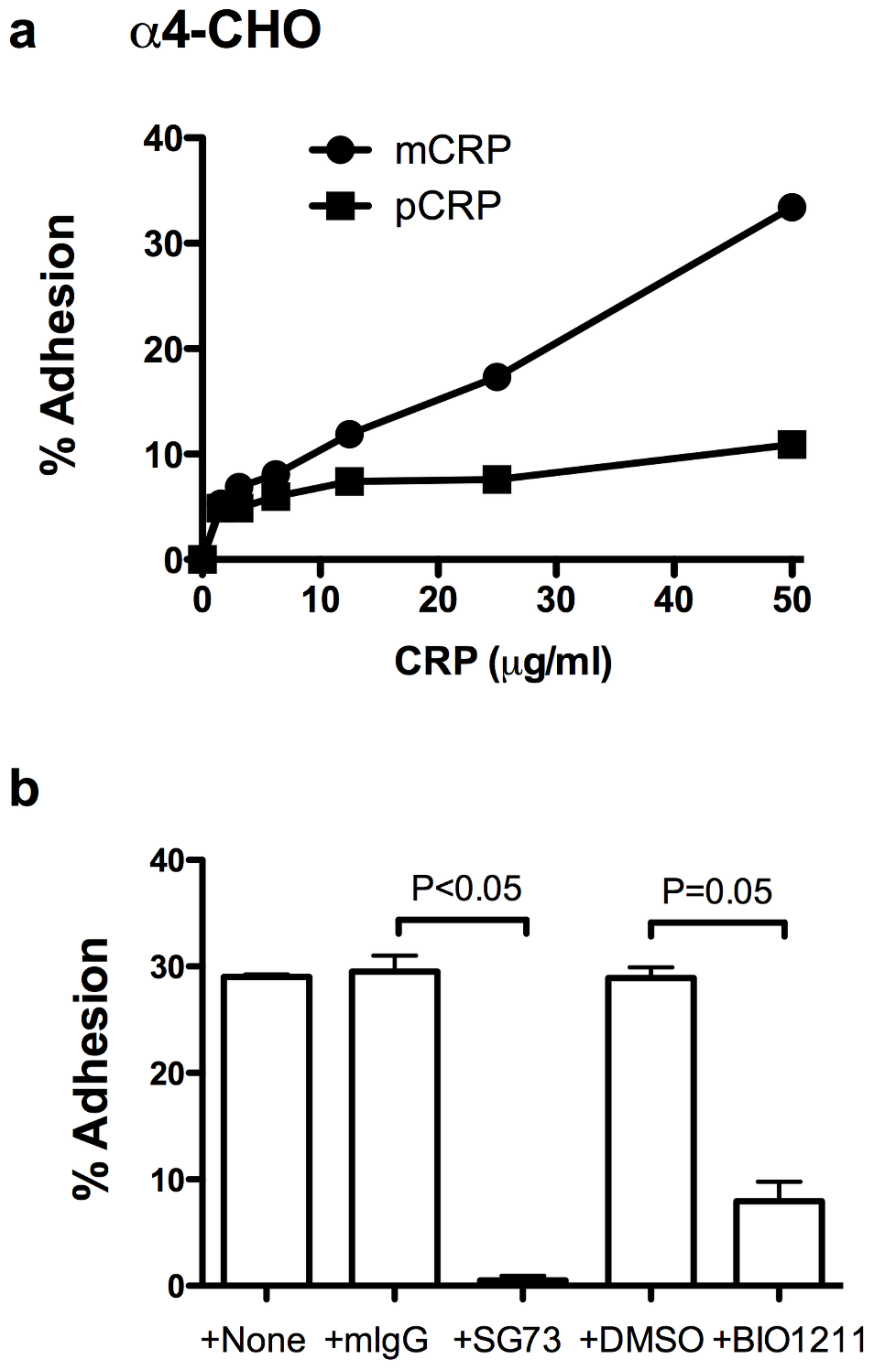
mCRP, and less efficiently pCRP, specifically bind to α4β1. a). Adhesion of α4-CHO cells to CRP isoforms. Wells of 96 well microtiter plate were coated with mCRP or pCRP and remaining protein-binding sites were blocked with BSA. Wells were incubated with CHO cells that express recombinant α4β1 (α4-CHO, 10^5^ cells per well) for 1 h in Tyrode-HEPES buffer with 1 mM MgCl_2_ and bound cells were quantified. b). Effect of antagonists to α4β1 on adhesion of α4-CHO cells to mCRP. Experiments were performed as in a). Fifty μg/ml coating concentration of mCRP was used. Antagonists were mAb SG73 (anti-human α4, 10 μg/ml) and BIO1211 (specific antagonist to α4β1, 1 μM). “mIgG” represents purified mouse IgG used as a control.

Integrin α4β1 is a major integrin expressed in immune competent cells [12]. We tested if CRP isoforms bind to integrin α4β1 using CHO cells that express recombinant human α4 (α4-CHO). We found that α4-CHO cells adhered to mCRP in a concentration-dependent manner and to a less extent to pCRP (Figure 3a). BIO1211, a specific inhibitor to α4β1 [24], and mAb SG73, a function-blocking anti-human α4, suppressed this interaction (Figure 3b). These findings suggest that integrins α4β1 is involved in binding to CRP isoforms as well. SG73 has been mapped in the ligand-binding site of α4 [25], suggesting that mCRP binds to the ligand-binding site of α4β1 as well.

### Integrins αvβ3 and α4β1 are involved in CRP binding in U937 monocytic cells

**Figure 4:**
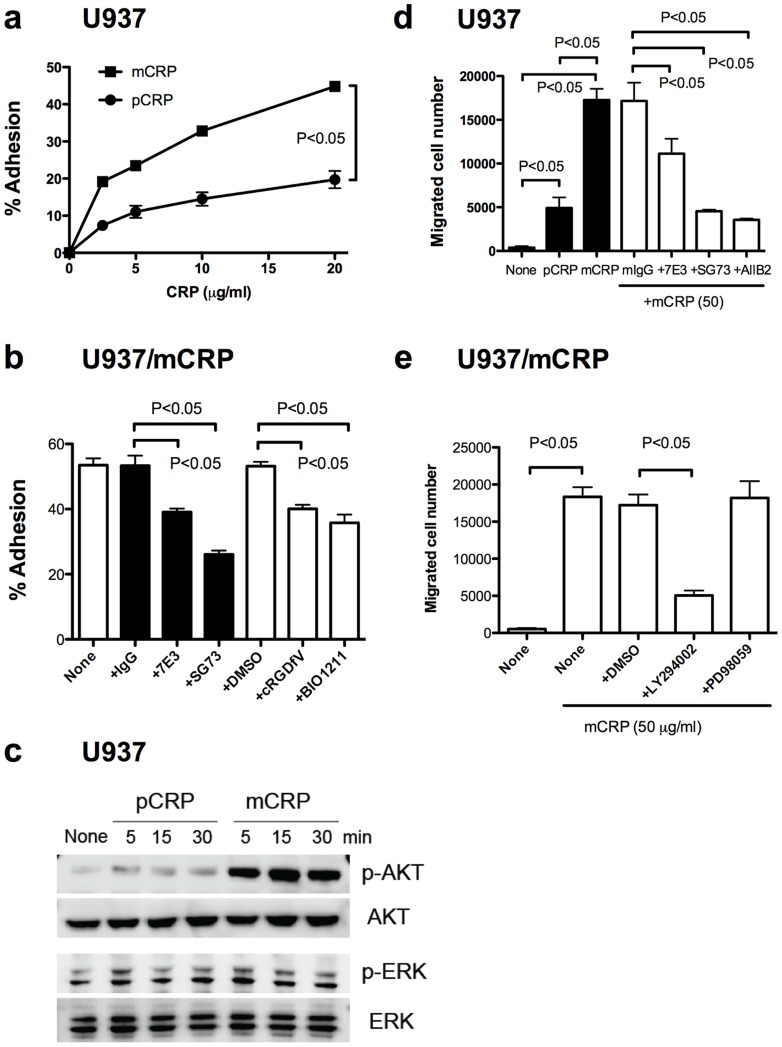
mCRP binds to U937 monocytic cells, and induces robust chemotaxis in an integrin-dependent manner. a). Adhesion of U937 cells to CRP isoforms. Wells of 96 well microtiter plate were coated with mCRP or pCRP and remaining protein-binding sites were blocked with BSA. Wells were incubated with U937 cells (10^5^ cells per well) for 1 h in RPMI1640 and bound cells were quantified. b) and c). Effect of antagonists to αvβ3 and α4β1 on adhesion of U937 cells to mCRP. In b), 2.5 μM coating concentration of mCRP was used. Antibodies used were mAb 7E3 (to human β3, 25 μg/ml), mAb SG73 (to human α4, 25 μg/ml), and AIIB2 (to human β1, 25 μg/ml). “mIgG” represents purified mouse IgG used as a control. Antagonists used were cyclic RGDfV (to αvβ3, 10 μM) and BIO1211 (to α4β1, 1 μM). DMSO was used as a control. Adhesion assay was performed in RPMI. d) mCRP induces AKT activation in U937 cells, but not ERK1/2 activation. U937 cells were serum-starved and stimulated with pCRP and mCRP (100 μg/ml) and cell lysates were analyzed by western blotting. d) mCRP, and less effectively pCRP, induce chemotaxis of U937 cells in an integrin-dependent manner. Chemotaxis was measured in modified Boyden chambers (Transwells). 50 μg/ml mCRP or pCRP in 600 μl RPMI 1640 medium was placed in the lower chamber, and U937 cells (5×10^5^ cells per well) were placed in the upper chamber. U937 cells were preincubated with antibodies (25 μg/ml) for 30 min at 37°C. After 4 h incubation, migrated cells were counted. e) A PI3K inhibitor, not MEK inhibitor, suppresses mCRP-induced chemotaxis of U937 cells. LY294002 (PI3K inhibitor) or PD98059 (MEK inhibitor) were added at 50 μM in the chemotaxis medium.

It has been reported that anti-CD16 antibody do not suppress the binding of mCRP to U937 cells [26] or human artery endothelial cells (HAEC) [27]. We thus studied if integrins αvβ3 and/or α4β1 play a role in mCRP binding in U937 cells. We found that U937 cells adhered to mCRP and to a less extent to pCRP in a concentration-dependent manner (Figure 4a). Antagonists specific to αvβ3 (mAb 7E3 and cRGDfV) and α4β1 (mAb SG73 and BIO1211) suppressed the adhesion of these cells to mCRP (Figs. 4b), suggesting that integrins αvβ3 and α4β1 are involved in mCRP binding in U937 cells.

We found that mCRP induced activation of AKT, while pCRP was less effective in this function (Figure 4c). We did not detect the effect of mCRP or pCRP on ERK1/2 activation (Figure 4c).

### mCRP induces chemotaxis of U937 cells in an integrin-dependent manner, but pCRP is much less effective in this function

It has been reported that mCRP, but not pCRP, can activate monocytes and can trigger events involved in rolling, adhesion, and finally transmigration [3]. We studied if mCRP-induced chemotaxis of U937 cells is dependent on mCRP binding to integrins in modified Boyden chamber. mCRP markedly induced chemotaxis of U937 cells, but pCRP induced it to a much less extent (Figure 4d). mAb against β3 (7E3), α4 (SG73), and β1 (AIIB2) effectively suppressed mCRP-induced chemotaxis (Figure 4d), suggesting that mCRP-induced chemotaxis is dependent on αvβ3 and α4β1. LY294002 (PI3K inhibitor) effectively suppressed mCRP-induced chemotaxis (Figure 4e), but PD98059 (MEK inhibitor) did not, which is consistent with the finding that mCRP induced AKT activation. These findings suggest that integrin binding and AKT activation are involved in mCRP-induced chemotaxis of U937 cells.

### Role of integrins in pro-inflammatory action of CRP isoforms

The present study establishes that integrins αvβ3 and α4β1 are novel receptors for mCRP and play a role in mCRP's pro-inflammatory action based on the following evidence. 1) Docking simulation predicts that mCRP binds to integrin αvβ3, but pCRP does not bind to αvβ3 due to steric hindrance. 2) mCRP bound to soluble and/or membrane-bound integrins αvβ3 and α4β1. The binding is specific since antagonists to αvβ3 or α4β1 suppressed the binding. The binding of pCRP to these integrins was at a much lower level. These findings suggest that αvβ3 and α4β1 are involved in the binding of mCRP and less effectively to pCRP. 3) mCRP induced AKT activation. 4) mCRP induced chemotaxis of U937 cells in an integrin-dependent manner, but pCRP was much less effective. Taken together, we propose that integrins act as receptors of mCRP and mediate pro-inflammatory actions of mCRP. It is highly likely that the dissociation of pCRP to mCRP exposes cryptic integrin-binding sites in CRP, and induces pro-inflammatory signaling through integrin and PI3K/AKT pathways. We propose that the enhanced binding of mCRP to integrins is related to its enhanced pro-inflammatory action.

As predicted by docking simulation, the integrin binding to pCRP was less effective than to mCRP. It has been reported that pCRP slowly dissociate into mCRP during storage [28]. Therefore it is possible that the weak binding of pCRP to integrins may be due to mCRP contamination in pCRP solution. To remove mCRP in pCRP solution, we treated the commercial pCRP preparation with phenyl-Sepharose, but the treatment did not reduce integrin binding to pCRP (data not shown). It is thus not likely that the weak interaction of pCRP with integrins may be due to mCRP contamination in pCRP preparations. It is likely that the integrin-binding sites are partially exposed in pCRP, but this may not be sufficient for pCRP to induce strong AKT activation or chemotaxis.

Integrin αvβ3 is highly expressed in endothelium and smooth muscle cells [29], and macrophages [22] in the atherosclerotic lesion. α4β1 is widely expressed in hematopoietic cells, including monocytes, macrophages, and neutrophils, and endothelial cells [1]. The present study directly connects pro-inflammatory action of mCRP and integrins αvβ3 and α4β1. It is also possible that mCRP binds to integrins other than αvβ3 and α4β1 in monocytic cells and other cell types. It is likely that integrins may mediate the binding of monocytic cells and other cell types to mCRP that has been deposited to the atherosclerotic region, and mediate pro-inflammatory signaling upon binding to mCRP. Also, it is possible that antagonists to these integrins exert anti-inflammatory actions through blocking mCRP-integrin interaction. To prove the importance of integrins in mCRP signaling, it would be necessary to use inhibitors that bind to mCRP and block integrin binding in future studies.
